# Cabbage Peptide miPEP156a Enhances the Level of Accumulation of Its mRNA in Transgenic Moss Physcomitrium patens

**DOI:** 10.32607/actanaturae.27668

**Published:** 2025

**Authors:** T. N. Erokhina, E. V. Ryabukhina, I. S. Lyapina, D. Y. Ryazantsev, S. K Zavriev, S. Y. Morozov

**Affiliations:** Shemyakin–Ovchinnikov Institute of Bioorganic Chemistry, Russian Academy of Sciences, Moscow, 117997 Russia; Lomonosov Moscow State University, Moscow, 119991 Russia

**Keywords:** microRNA, peptides, miPEP, pri-miRNA, transgenic plants, PCR analysis

## Abstract

MicroRNAs are endogenous, small non-coding RNAs that regulate gene expression at the post-transcriptional level by cleaving target mRNAs. Mature microRNAs are products of the processing of their primary transcripts (pri-miRNAs). Now, it has been discovered that the products of the translation of some plant pri-miRNAs are peptide molecules (miPEP). These peptides have the capacity to physically interact with their open reading frames (ORFs) in the transcribed pri-miRNAs and, thus, positively regulate the accumulation of these RNAs and the corresponding mature microRNAs. Most conserved microRNAs play an important role in plants development and their response to stress. In this work, we obtained transgenic* Physcomitrium patens *moss plants containing *Brassica oleracea *miPEP156a ORF in the genome under the control of a strong 35S cauliflower mosaic virus promoter and analyzed the effect of the exogenous peptide on the transcription of this ORF in the protonemata of two transgenic moss lines. It turned out that the chemically synthesized peptide miPEP156a increases the accumulation of its own mRNA during moss culture growth, as was previously shown in studies by foreign researchers and in our own work for a number of peptides in monocotyledonous and dicotyledonous plants. These findings confirm that pri-miRNA regions that are located outside the coding region of the peptide are not required for transcriptional activation. Moreover, we have also succeeded in showing that the presence of a specific promoter of the microRNA gene does not affect the phenomenon of transcription activation; this phenomenon *per se *is not species-specific and is observed in transgenic plants, regardless of the origin of the miPEP.

## INTRODUCTION

MicroRNA genes are known to be transcribed in the form of large primary transcripts (pri-miRNA) and to become mature miRNAs only after several maturation stages [1]. Like any protein-coding gene, microRNA genes are transcribed by RNA polymerase II, yielding the primary transcript pri-miRNA, which consists of several hundreds or thousands of nucleotides. The internal domain of this primary transcript contains a characteristic hairpin structure consisting of a partially double-stranded microRNA sequence which is cleaved into its mature form under the action of the DCL1 protein encoded by the *Dicer *gene [1]. First, this enzyme cleaves the 5’- and 3’ terminal regions of the primary transcript to convert the transcript into a hairpin-like miRNA precursor (pre-miRNA) and, then, cleaves pre-miRNA to release the miRNA-miRNA* duplex. This duplex is then translocated into the cytoplasm, where one of the strands (corresponding to microRNA) is incorporated into the ribonucleoprotein particle formed by Argonaute nuclease, giving rise to the RISC complex, which further ensures microRNA-mediated gene silencing [1].

Ten years ago, a number of pri-miRNAs were found to contain small open reading frames which can encode regulatory peptides known as microRNAencoded peptides (miPEPs) [2]. In plants, miPEPs potentiate the transcription and accumulation of the respective pri-miRNA, being an example of positive feedback. This enhances the accumulation of mature microRNAs and suppression of the target genes for microRNAs [3, 4]. Overexpression of miPEP in the treatment of leaves and roots with chemically synthesized exogenic peptides may significantly alter the development of roots, as well as enhance anthocyanin accumulation and resistance to biotic and abiotic stress [4, 5, 6, 7]. Importantly, a number of these phenomena can be successfully used to improve the commercially significant properties of plants [4, 5, 7, 8].

Previously, we employed the bioinformatic approach to perform a comparative analysis of ORF sequences within pri-miRNA genes in plant genomes and identified a novel group of miPEPs (miPEP156a peptides) encoded by pri-miR156a across several dozen species belonging to the *Brassicaceae *family [9]. Chemically synthesized exogenous miPEP156a peptides can efficiently penetrate plant seedlings through their root system and systemically migrate to leaves. The peptides exhibit an explicit morphological effect that accelerates primary root growth. Simultaneously, miPEP156a peptides upregulate the expression of their own pri-miR156a [9]. Importantly, this peptide is able to rapidly enter the cell nucleus and bind to chromatin. In this study, we have identified the general properties of the secondary miPEP156a structure and detected its alterations that are induced by the formation of a peptide complex with nucleic acids [9].

It has been proved recently that the Mt-miPEP171b peptide expressed in legumes interacts with transcribed (mostly incomplete) pri-miR171b molecules within the complex with the chromosomal DNA template strand. A hypothesis has been put forward that this is a novel type of protein–RNA binding that is entirely dependent on the presence of a specific linear codon set in the template encoding this miPEP, and that these peptides can perform specific regulatory functions only with respect to their pri-miRNAs [10]. Based on the features of the interaction between miPEPs and their pri-miRNA, the following sequence for the activation of pri-miRNA transcription by encoded peptides has been proposed: (1) in the cytoplasm, miPEP is translated from full-length pri-miRNA or its fragment comprising the ORF encoding the miPEP; (2) this peptide then migrates to the nucleus, where it binds to the synthesized pri-miRNA within its coding sequence; and (3) this interaction boosts microRNA accumulation at the transcriptional level [4, 10]. Existing experimental evidence precludes drawing any conclusion as to whether miPEP binds to the ribonucleotide strands of pri-miRNA (or RNA, forming an RNP) or to RNA–DNA hybrids. Clearly, it cannot be ruled out that miPEPs can interact not only with RNA, but also, as demonstrated previously [3], with DNA (and/or chromatin) in microRNA gene regions. Hence, miPEPs can regulate the activity of RNA polymerase II and/or the mediator complex during the transcription initiation and/or elongation stage [8].

Our study, employing the model of miPEP156a expressed in cabbage (*Brassica oleracea*), aimed to elucidate the following: (1) the significance of the primiRNA regions outside the peptide-coding domain for transcriptional activation; (2) the role of the specific microRNA gene promoter in the transcriptional activation phenomenon; and (3) whether the transcriptional activation phenomenon is species-specific: i.e., whether the miPEP peptide from one plant species can function in other taxonomically distant species. For this purpose, we engineered transgenic moss (*Physcomitrium patens*) plants harboring the ORF for broccoli miPEP156a in their genome, under the control of the strong cauliflower mosaic virus (CaMV) 35S promoter, and analyzed the effect of the exogenous peptide on the transcription of this ORF in moss protonemata.

## EXPERIMENTAL PART

**Fig. 1:**
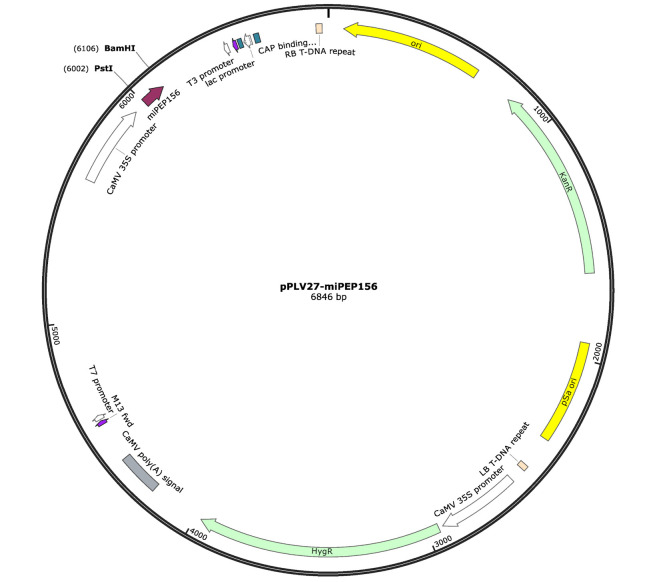
Scheme of plasmid pPLV27-miPEP156a bearing the coding region of miPEP156a (shown with a violet arrow) for expression in transgenic plants

The coding region of the miPEP156a ORF, including the initiation and termination codons, was amplified by polymerase chain reaction (PCR) using the chromosomal DNA from *B. oleracea *as a template. A pair of DNA primers was used for this purpose: mir156r (5’-CTTTCTTTATGGCTCTTGTCGCTT) and mir156f (5’-AAATGTTCTGTTCAATTCAATGC) [9]. The resulting amplification product was cloned into the pPLV27 vector using ligation-independent cloning (LIC) [11]. Following cloning, the pPLV27-miPEP156a plasmid (*Fig. 1*) was propagated in *Escherichia coli* cells, purified using the Qiagen Plasmid Maxi Kit (Qiagen, Germany), and sequenced.

To engineer transgenes, protonemata of the *P. patens* moss (Gransden 2004 strain) were cultivated on 9-cm Petri dishes containing a solid Knop medium supplemented with 1.5% agar (Helicon, Russia) and 500 mg/L ammonium tartrate (Helicon) under white light illumination from fluorescent lamps (MLR-352H Sanyo Plant Growth Incubator, Panasonic, Japan), with a photon flux density of 61 μmol/m2 under a 16-h photoperiod at 24°C and 50% relative humidity. Protoplasts were isolated from five-day-old protonemal tissue. The protonemata were collected from the agar surface using a spatula, gently pressed, and placed into a 0.5% Driselase solution (Sigma-Aldrich, USA) in 0.48 M mannitol for 45 min under continuous rocking in the dark. The resulting suspension was filtered through a 100-μm metal sieve. The protoplasts were pelleted in 50-mL plastic tubes by 5-min centrifugation at 150 g and washed twice with 0.48 M mannitol, followed by centrifugation under identical conditions. After removing the supernatant, the protoplasts were transformed according to the PEGmediated transformation protocol in [11]. The protoplasts (1.5 × 106/mL) were resuspended in a MMg solution (0.48 M mannitol, 15 mM MgCl_2_, 0.1% MES, pH 5.6) and incubated for 20 min. Next, 10 μg of the pPLV27-miPEP156a plasmid (*Fig. 1*) and 33% PEG solution were added, followed by an additional 30 min of incubation. After washing, the protoplasts were plated in top agar in Petri dishes containing a filmcoated solid medium. The plates were kept in the dark for 24 h and then transferred to standard cultivation conditions to allow protoplast regeneration.

**Fig. 2:**
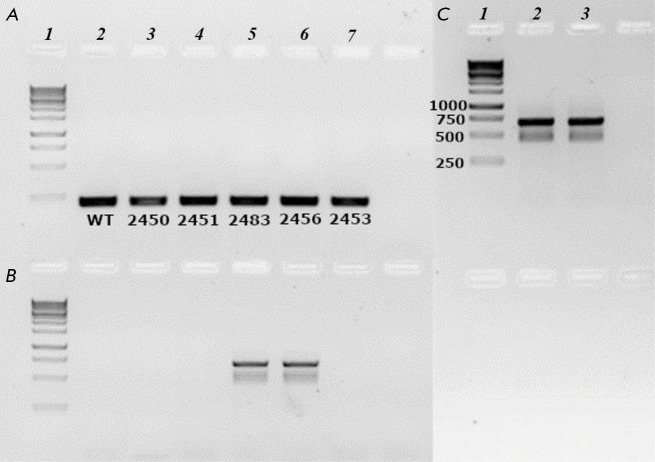
Results of PCR for detection of the desired inserts in the selected hygromycin- resistant lines of *P. patens *(lines 2450, 2451, 2483, 2456, and 2453). Genomic DNA from the non-transformed moss line (WT) was used as the control. (*A*) – control PCR for reference moss gene EF1-alpha (translation elongation factor 1a). Lane *1 *– DNA size markers; *2 *– WT;* 3 *– line 2450; *4 *– line 2452; *5 *– line 2483; *6* – line 2456; and *7 *– line 2453. (*B*) – PCR of moss genomic DNA with the primers p35Sf and tNOSr. Lanes are arranged identically to panel (*A*). (*C*) – second PCR experiment with slow-growing moss line 2450. Lane *1 *– DNA size markers; *2 *– line 2550; and *3 *– DNA of plasmid pPLV27-miPEP156a

To select the clones carrying the target gene insertion, the regenerated protoplasts were cultivated on a selective medium containing hygromycin. Five stable transgenic lines of *P. patens *moss were identified: 2450, 2451, 2453, 2456, and 2483. Total DNA was extracted from the plant tissues of these lines using a DNeasy Plant Kit according to the manufacturer’s protocol (Qiagen). The PCR analysis shows that specific reaction products carrying a 700-bp miR156a ORF insertion, obtained using the primers p35Sf (5’-AACAAAGGATAATTTCGGGAAAC) and tNOSr (5’-TCGCGTATTAAATGTATAATTGC) complementary to the pPLV27 plasmid regions carrying the 35S promoter and transcription terminator, respectively (*Fig. 1*), formed only in lines 2450 and 2483 (*Fig. 2*). Insertion specificity was confirmed by sequencing the PCR products. Interestingly, the colonies of these moss lines were characterized by substantially different growth rates. Whereas the growth rate of line 2483 was similar to that of wild-type plants, slower colony development was observed for line 2450. Therefore, it is important to mention that transgenic* P. patens *plants overexpressing a number of endogenous peptides also tend to exhibit a reduced colony growth rate [11].

**Fig. 3:**
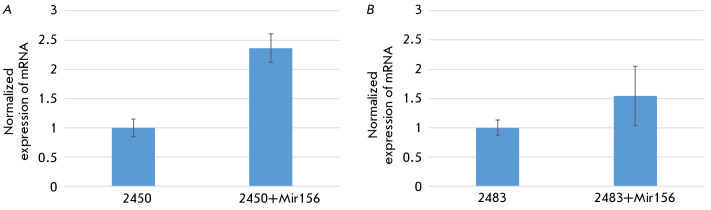
PCR for measuring the expression level for mRNA of miPEP156a in the transgenic moss *P. patens *lines. (*A*) line 2450 and (*B*) line 2483. RNA was isolated from five independent moss cultures for each line. The figure shows the average statistical values as a bar chart and standard deviations. The statistical significance of the differences in the sum of values in the control experiments (without incubation with the peptide) and experimental experiments (with miPEP156a peptide added) was *p* < 0.05 for these two samples according to the Student’s t test (GraphPad Prism 7.0, https://graphpad_prism.software.informer.com/7.0/)

To investigate the effect of the miPEP156a peptide on mRNA expression in transgenic plants by quantitative PCR, protonemata of lines *P. patens *2450 and 2483 were cultured in 100 mL of a liquid Knop medium supplemented with 500 mg/L ammonium tartrate (Helicon, Russia) on rocking shakers under white light illumination from fluorescent lamps in a Sanyo Plant Growth Incubator MLR-352H (Panasonic, Japan), with a photosynthetic photon flux density of 61 μmol/m2, under a 16-h photoperiod at 24°C and 50% relative humidity. For the analysis, seven-day-old protonemata were treated with an aqueous solution of the peptide (5 μg/mL) in a final volume of 50 mL. The samples were incubated overnight. Next, the protonemal filaments were separated from the medium, blotted using filter paper to remove excess moisture, and flash-frozen in liquid nitrogen. Total RNA was extracted from the frozen tissues using the TRIzol™ reagent (Invitrogen, USA) according to the manufacturer’s protocol. After concentration quantification, 2 μg of RNA was treated with DNase I (Thermo Fisher Scientific, USA). Reverse transcription with a random hexamer primer using a Mini kit (Eurogen, Russia) was then performed. The resulting cDNA was added to the qPCR reaction mixture using the reagents and protocols provided by the manufacturer (Eurogen). Quantitative PCR was carried out using the premixed qPCRmix HS (Eurogen) on a DTprime amplification system (DNA-Technology, Russia). The reaction mixture (25 μL) contained 10 pmol of each primer and 1× Eva Green intercalating dye. The following amplification program was used: 95°C – 5 min; 95°C – 15 s, 60°C – 15 s^*^; 72°C – 15 s; 45 cycles (* – fluorescence detection in the FAM channel). After data processing, the Cq values were used to calculate normalized expression using the QGene software [12] (*Fig. 3*).

## RESULTS AND DISCUSSION

Quantitative PCR (*Fig. 3*) revealed that treatment of the moss culture with chemically synthesized exogenous peptide miPEP156a in the nutrient solution enhanced the accumulation level of its own mRNA in the transgenic moss culture. In line 2483, accumulation of the RNA template for the miPEP156a peptide increased by ~ 60%. This effect was even more pronounced for line 2450: accumulation of the RNA template for the cabbage peptide increased by 240%, exceeding the effect observed for treated cabbage seedlings [9].

In this study, we generated transgenic moss* P. patens *plants harboring the ORF for broccoli miPEP156a in their genome under the control of the strong Cauliflower Mosaic Virus 35S promoter and analyzed the effect of the exogenous peptide on the transcription of this ORF in the protonemata of two transgenic moss lines. The chemically synthesized exogenous miPEP156a peptide was found to enhance the accumulation of its own mRNA in the moss culture, as was demonstrated previously for a range of peptides in monocotyledonous and dicotyledonous plants [3, 4, 5]. Our findings here indicate that pri-miRNA regions outside the peptide-coding domain are not required for transcriptional activation. Furthermore, the specific microRNA gene promoter is not involved in the transcriptional activation phenomenon, and activation *per se *is not species-specific. In other words, the miPEP156a peptide expressed in* Brassica *plants can function in other taxonomically distant plant species such as moss. Hence, our findings are consistent with the proposed mechanism of miPEP action, where the peptide binds to its own transcribed pri-miRNA template, thereby activating the synthesis of these RNAs [10].

## Abbreviations

pri-miRNA: primary transcripts of microRNA genes
miPEP: peptide encoded by primary transcript of microRNA genes
OTF: open translation frame
PCR: polymerase chain reaction
